# Mucinous Cystadenoma of the Kidney: A Rare Case and Literature Review

**DOI:** 10.7759/cureus.108706

**Published:** 2026-05-12

**Authors:** Muhammed N Karadeniz, Mehmet Demir, Hasan Otlu, Muhammed Almuhammed, Ferhat Coskun

**Affiliations:** 1 Department of Urology, Harran University Hospital, Sanlıurfa, TUR; 2 Department of Pathology, Sanlıurfa Education and Research Hospital, Sanlıurfa, TUR

**Keywords:** bosniak type ııı cyst, cystadenocarcinoma, kidney, mucinous cystadenoma, nephrectomy

## Abstract

Mucinous cystic tumors of the renal pelvis or pyelocaliceal system are extremely rare neoplasms and often mimic complicated renal cysts. Surgical resection is recommended due to the risk of malignant transformation.

In September 2023, imaging revealed a cystic lesion measuring 106×93 mm in the right kidney of a 68-year-old male patient, reported as Bosniak type III. The patient underwent right radical nephrectomy. Pathological examination revealed a unilocular cystic lesion lined with mucinous epithelium showing no atypia. Immunohistochemically, epithelial cells were CDX2 positive and PAX8, GATA3, and p63 negative. Findings were consistent with mucinous cystadenoma. No recurrence or metastasis was observed during 18 months of follow-up.

The diagnosis of renal mucinous cystadenoma is challenging with preoperative imaging, and the definitive diagnosis is made by postoperative histopathology. Surgical resection and close follow-up are necessary due to the risk of malignant transformation.

## Introduction

Mucinous cystic tumors arising from the renal pelvis or pyelocaliceal system are extremely rare and may radiologically mimic complex renal cysts [1]. Accurate diagnosis is challenging, and these lesions may be misinterpreted as malignant cystic renal tumors [2].

This case highlights the diagnostic difficulty of renal mucinous cystadenoma on preoperative imaging and emphasizes the importance of histopathological and immunohistochemical evaluation in differentiating it from malignant renal neoplasms. Reporting such rare cases contributes to improved recognition and management of complex cystic renal lesions [3].

## Case presentation

An asymptomatic 68-year-old male with a history of chronic arterial disease treated with clopidogrel was found to have a palpable mass in the right upper quadrant on physical examination. Laboratory evaluation showed a glomerular filtration rate (GFR) of 77 mL/min/1.73 m² and a serum creatinine level of 1.00 mg/dL.

**Figure 1:**
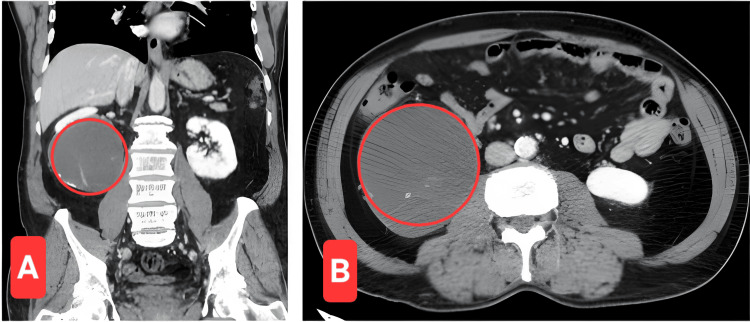
Contrast-enhanced abdominal CT shows a renal lesion circled in red in both coronal (A) and axial plane (B)

In September 2023, routine imaging identified a cystic lesion in the right kidney. Dynamic contrast-enhanced abdominal computed tomography (CT) demonstrated a Bosniak type III cystic lesion measuring 106 × 93 mm, originating from the lower pole of the right kidney, with peripheral calcifications and thick septa. Renal parenchymal atrophy secondary to compression by the cystic lesion was observed. No extrarenal involvement or distant metastasis was detected (Figure 1).

Given the large size of the lesion, associated parenchymal atrophy, and suspicion of malignancy, an open right radical nephrectomy was performed.

**Figure 2:**
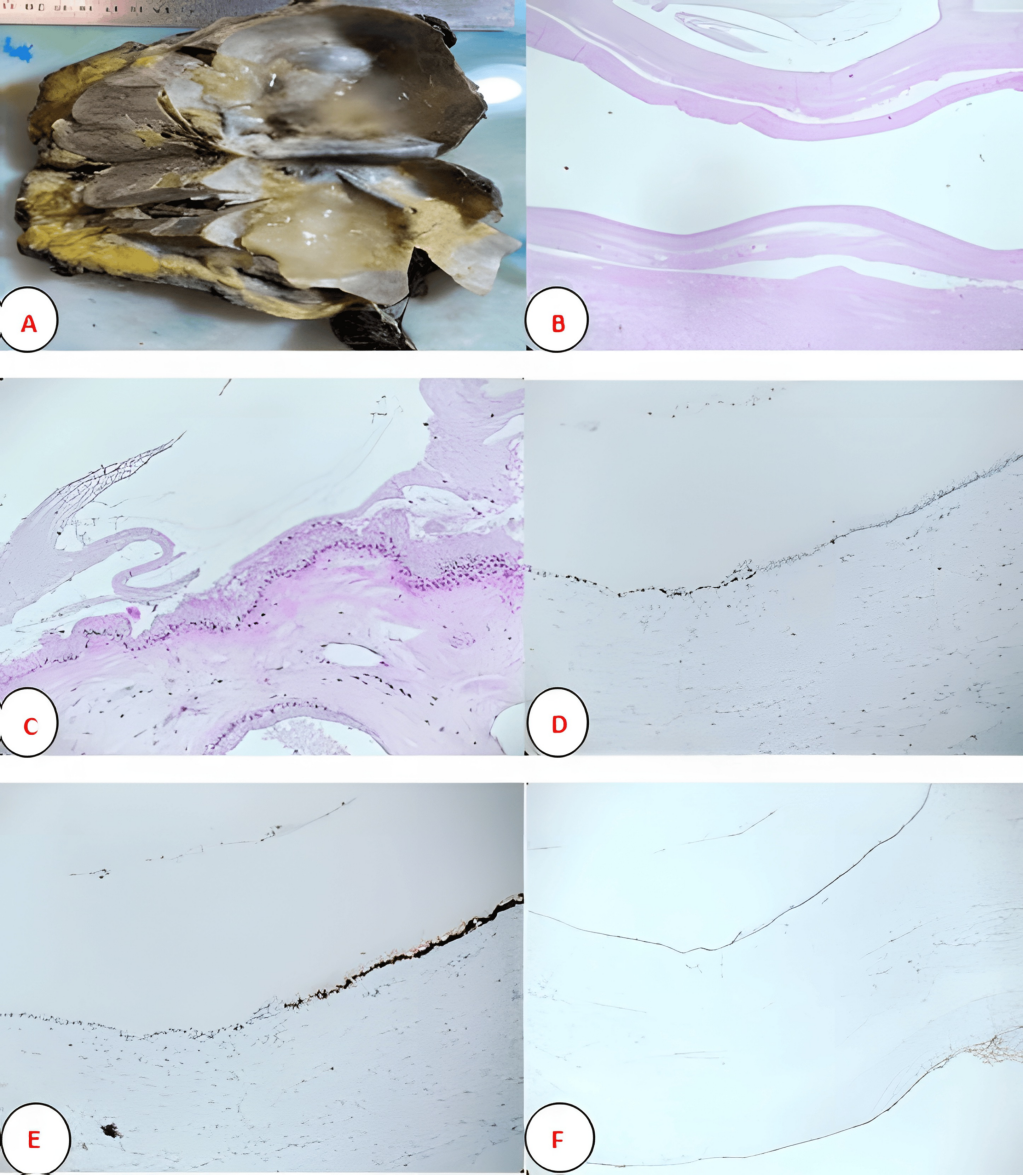
Pathology specimen images A; Macroscopic appearance of the specimen, B; 4x10 objective, cyst wall lined with mucinous epithelium, collecting duct lined with urothelial epithelium in between, and renal parenchyma below C; 20x10 objective, cyst wall lined with mucinous epithelium without single-row atypia, D; 20x10 objective, nuclear p63 positive in urothelial epithelium observed in continuation of mucinous epithelium, E; 20x10 objective, nuclear CDX2 positive observed in mucinous epithelium, F; 10x10 objective, urothelial epithelium showing nuclear positivity with GATA3 in the continuation of the mucinous epithelium and in the underlying duct, mucinous epithelium GATA3 negative.

Gross pathological examination revealed a unilocular cyst containing mucoid fluid. Microscopic examination demonstrated that the cyst wall was lined by mucinous epithelium without cytological atypia. Immunohistochemical staining showed positivity for CDX2 and negativity for PAX8, GATA3, and p63. These findings supported the diagnosis of mucinous cystadenoma (Figure 2).

Follow-up included ultrasonography every six to 12 months, clinical evaluation, and laboratory assessment. At 18 months, no recurrence or metastasis was observed. Laboratory results demonstrated a GFR of 55 mL/min/1.73 m² and a serum creatinine level of 1.45 mg/dL.

## Discussion

Mucinous tumors are most commonly encountered in the appendix and ovaries; however, no definitive relationship has been established between renal mucinous cystadenoma and tumors arising from these organs [4-6]. To date, only 21 cases of renal mucinous cystic neoplasms have been reported, four of which were cystadenocarcinomas (Table 1) [7].

**Table 1:** Clinical and pathological features of 21 mucinous cystic neoplasms of the pyelocaliceal system. Abbreviations: CT, carcinoid tumor; HGD, high-grade dysplasia; IMCa, intramucosal carcinoma; MCN, mucinous cystic neoplasm; MT, malignant transformation; MuC, mucinous cystadenoma; MCa, mucinous cystadenocarcinoma; NS, not specified

Case number	Gender	Age	Anatomical location	Size (cm)	MCN Type	Stone disease	Ref
1	Male	79	Right	3.5	MuC	No	[7]
2	Male	31	Right	Not specified	MuC	Not specified	[2]
3	Female	35	Left	Not specified	MuC	No	[8]
4	Male	65	Left	13	MuC	Yes	[8]
5	Male	63	Right	21	MuC	Yes	[9]
6	Female	27	Horseshoe	12	MuC	No	[10]
7	Female	59	Horseshoe	7	MuC	Unspecified	[11]
8	Male	50	Right	4.5	CT with MuC	No	[12]
9	Female	53	Right	27	CT with MuC	Yes	[13]
10	Male	64	Right	37	MuC	Yes	[14]
11	Male	54	Left	20	MuC	Yes	[14]
12	Female	63	Right	Not specified	HGD/Borderline with MuC	Yes	[15]
13	Male	69	Right	5	MT with MuC	No	[16]
14	Female	62	NS	Not specified	MT with MuC	Not specified	[17]
15	Female	63	Right	Not specified	HGD/Borderline with MuC	Yes	[18]
16	Male	52	Left	35	IMCa with MuC	Yes	[19]
17	Male	45	Left	2.4	IMCa and MuC	No	[14]
18	NS	NS	NS	30	MCa	Yes	[20]
19	Male	45	Left	30	MCa	Yes	[21]
20	NS	NS	Not specified	Not specified	MCa	Not specified	[22]
21	NS	NS	Not specified	Not specified	MCa	Not specified	[23]

Primary renal mucinous cystadenomas are rare and may arise either from the renal pelvis or parenchyma [8,10]. In our case, histopathological examination demonstrated continuity between the mucinous epithelium and adjacent urothelium, supporting a pelvic origin.

Preoperative diagnosis remains challenging, and none of the previously reported cases were correctly diagnosed based on imaging alone [24]. The immunohistochemical profile observed in our patient - CDX2 positivity with PAX8, GATA3, and p63 negativity - was crucial in confirming the diagnosis and excluding both conventional renal cell carcinoma and metastatic mucinous tumors of non-renal origin [25].

Although malignant transformation has been reported in a minority of cases, metastasis from a histologically confirmed benign renal mucinous cystadenoma has not been documented to date [4-6]. Radical or partial nephrectomy remains the standard treatment because of diagnostic uncertainty and the potential for malignant transformation.

Histopathological examination of the specimen revealed the continuity of the mucinous epithelium close to the urothelium, suggesting that the tumor originated from the renal pelvic epithelium. One proposed mechanism is intestinal metaplasia of the urothelium secondary to chronic irritation. It has been suggested that mucinous tumors of the renal pelvis may be associated with urinary system stones and that chronic inflammation of the epithelium may be a possible factor in their development [26]. Another view argues that stone formation develops secondary to mucin secretion from the cystic tumor [14]. Although many previously reported cases had accompanying kidney stones, our patient had no urinary system stones.

Our case further supports the concept that renal mucinous cystadenoma should be considered in the differential diagnosis of large Bosniak III cystic renal lesions.

Long-term follow-up is advisable in these rare tumors to better understand their biological behavior. A limitation of our report is the relatively short follow-up period of 18 months, which may not fully reflect long-term recurrence risk.

## Conclusions

This case demonstrates that renal mucinous cystadenoma may present radiologically as a Bosniak type III complex cyst and can be indistinguishable from malignant cystic renal tumors preoperatively. Definitive diagnosis relies on histopathological and immunohistochemical evaluation. In large or suspicious lesions with parenchymal compromise, radical nephrectomy may be an appropriate therapeutic approach. Careful postoperative surveillance is recommended despite the generally benign course.
